# Tannic Acid and Ethyl Gallate Potentialize Paclitaxel Effect on Microtubule Dynamics in Hep3B Cells

**DOI:** 10.3390/ph16111579

**Published:** 2023-11-08

**Authors:** Jessica Nayelli Sánchez-Carranza, Mariano Redondo-Horcajo, Isabel Barasoain, Ever Angel Escobar-Aguilar, César Millán-Pacheco, Laura Alvarez, Enrique Salas Vidal, J. Fernando Diaz, Leticia Gonzalez-Maya

**Affiliations:** 1 Facultad de Farmacia, Universidad Autónoma del Estado de Morelos, Av. Universidad 1001, Cuernavaca 62209, Morelos, Mexico; jessica.sanchez@uaem.mx (J.N.S.-C.); ever.escobaraug@uaem.edu.mx (E.A.E.-A.); cmp@uaem.mx (C.M.-P.); 2 Centro de Investigaciones Biológicas Margarita Salas—Consejo Superior de Investigaciones Científicas, 28040 Madrid, Spain; marecib@cib.csic.es (M.R.-H.); barasoain.isabel@gmail.com (I.B.); 3 Centro de Investigaciones Químicas-IICBA, Universidad Autónoma del Estado de Morelos, Av. Universidad 1001, Cuernavaca 62209, Morelos, Mexico; lalvarez@uaem.mx; 4 Departamento de Genética del Desarrollo y Fisiología Molecular, Instituto de Biotecnología, Universidad Nacional Autónoma de México, Cuernavaca 62209, Morelos, Mexico; esalas@ibt.unam.mx

**Keywords:** paclitaxel, tannic acid, ethyl gallate, tubulin, cancer, hepatocellular carcinoma, microtubules

## Abstract

Among broad-spectrum anticancer agents, paclitaxel (PTX) has proven to be one of the most effective against solid tumors for which more specific treatments are lacking. However, drawbacks such as neurotoxicity and the development of resistance reduce its therapeutic efficacy. Therefore, there is a need for compounds able to improve its activity by synergizing with it or potentiating its effect, thus reducing the doses required. We investigated the interaction between PTX and tannins, other compounds with anticancer activity known to act as repressors of several proteins involved in oncological pathways. We found that both tannic acid (TA) and ethyl gallate (EG) strongly potentiate the toxicity of PTX in Hep3B cells, suggesting their utility in combination therapy. We also found that AT and EG promote tubulin polymerization and enhance the effect of PTX on tubulin, suggesting a direct interaction with tubulin. Biochemical experiments confirmed that TA, but not EG, binds tubulin and potentiates the apparent binding affinity of PTX for the tubulin binding site. Furthermore, the molecular docking of TA to tubulin suggests that TA can bind to two different sites on tubulin, one at the PTX site and the second at the interface of α and β-tubulin (cluster 2). The binding of TA to cluster 2 could explain the overstabilization in the tubulin + PTX combinatorial assay. Finally, we found that EG can inhibit PTX-induced expression of pAkt and pERK defensive protein kinases, which are involved in resistance to PXT, by limiting cell death (apoptosis) and favoring cell proliferation and cell cycle progression. Our results support that tannic acid and ethyl gallate are potential chemotherapeutic agents due to their potentiating effect on paclitaxel.

## 1. Introduction

The International Agency for Research on Cancer estimated that by 2020, liver cancer would be the seventh leading cause of cancer incidence and the fourth leading cause of cancer mortality across all ages and sexes worldwide. The majority of primary liver tumors (more than 90%) are hepatocellular carcinomas (HCC). These tumors have similar incidence and mortality rates, with a 5-year survival rate of 19.8% in the US and less than 3% if diagnosed at an advanced metastatic stage [1]. In the latter case, there is no effective long-term treatment. Systemic therapy with tyrosine kinase inhibitors (TKIs) becomes the most viable option. However, the tumors of most HCC patients become resistant to this chemotherapy within a few months, making the development of new treatments capable of targeting this tumor of great interest. Tumors with or without specific treatments can benefit from broad-spectrum antitumor drugs, including paclitaxel (PTX) and its analogue docetaxel, which have shown benefits in many solid tumor types. PTX is a potent anticancer drug that binds to microtubules, stabilizing them and inhibiting microtubule depolarization during mitosis [2,3].

Systemic PTX chemotherapy, either alone or in combination, has also been shown to be effective in the treatment of ovarian, breast, lung, head, and neck cancers, including HCC [4,5,6,7,8,9]. However, there are significant disadvantages associated with PTX chemotherapy, such as peripheral neurotoxicity and the development of clinical resistance, leading to chemotherapeutic failure, disease progression, and, ultimately, patient death. Tumor resistance to PTX involves several mechanisms, including efflux pump modification, epithelial–mesenchymal transition (EMT), kinase-dependent cyclin 1 (CDK1), the cell death signaling pathway, and finally, the overexpression of a less PTX-sensitive β-tubulin isotype β-III, which has been consistently reported in many types of acquired PTX-resistant cancers [10,11,12,13]. Furthermore, PTX promotes the phosphorylation and, therefore, the activation of pAkt and pErk, proteins involved in chemoresistance, suggesting that the AKT and ERK pathways are potential therapeutic targets to overcome resistance to PTX [14,15,16].

Therefore, potentiating PTX activity or modulating resistance is a critical issue in cancer therapy. Compounds capable of reversing or reducing PTX resistance in cancer cells (chemosensitizers) can significantly improve treatment efficacy and patient response. In this sense, many polyphenolic compounds in dietary plants have anticancer and chemosensitizing properties [17,18]. Among these, the phenolic compounds tannic acid (TA) and ethyl gallate (EG), found in many plants, have shown cytotoxic effects, such as reduction in cell viability, induction of cell cycle arrest, and even induction of cell death in several cancer lines [19,20,21].

In a previously published study, we demonstrated that TA and EG reduced cell viability, induced G2/M cell arrest, and promoted apoptosis in Hep3B and HepG2 HCC cells. Remarkably, we observed that TA and EG stabilized microtubules similar to PTX. Furthermore, TA and EG did not show a relevant cytotoxic effect on immortalized liver cells (IHH) used as a non-cancer cell control [22].

Aware of the antitumor effects of TA or EG with PTX and other chemotherapeutic drugs, which can improve and/or complement conventional treatments, we carried out this study, focusing on the effect of TA and EG on microtubule stabilization and on the combinatorial effect with PTX; we performed cellular, biochemical, molecular, and in silico analyses, both in Hep3B cells and in purified tubulin, to elucidate the mechanism of this effect.

## 2. Results

### 2.1. TA and EG Treatment Enhance the Inhibitory Effect of PTX on the Viability of Hep3B HCC Cancer Cells

**Figure 1:**
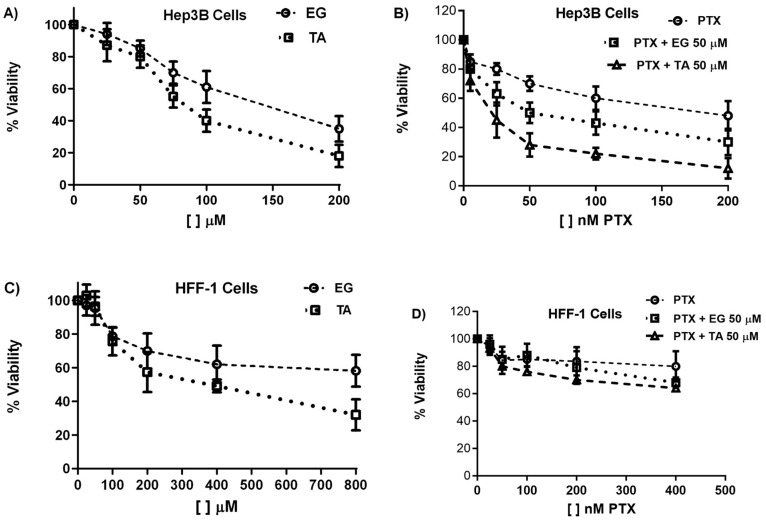
Effect of EG, TA, and PTX on Hep3B cell viability. (**A**) Effect of different concentrations of EG and TA on Hep3B cell viability. (**B**) Effect of different concentrations of PTX alone and in combination with 50 μM EG or 50 μM TA on Hep3B cell viability. (**C**) Effect of different concentrations of EG and TA on HFF-1 cell viability. (**D**) Effect on cell viability of different concentrations of PTX alone and in combination with 50 μM EG or 50 μM TA on HFF-1 cells. Absorbance values are expressed relative to drug-free culture cells (arbitrary value of 100). Results are the mean ± SD of at least three independent experiment repetitions.

First, we evaluated the effect of TA and EG individually at concentrations of 5, 25, 50, 100, and 200 μM. We observed that at 50 μM TA or EG, there was between 85 and 90% cell viability. This concentration was chosen at which, individually, there was no significant effect on cell viability after 24 h of treatment (Figure 1A) in order to rule out the possibility that the observed effect was due to the cytotoxic effect of each compound by itself (Figure 1A). Subsequently, we evaluated the combined effect of TA or EG at 50 μM with increasing concentrations of PTX (5, 25, 50, 100, and 200 nM) to determine whether the combination would enhance the effect of PTX on cell viability (Figure 1B). The cell viability results showed a more significant effect of the combination of PTX with TA or EG compared to the single effect of PTX. The combined effect of PTX with EG decreased the viability of Hep3B cells; however, the combined effect with TA was more substantial (Figure 1B).

We also evaluated cell viability in human fibroblast cells, HFF-1. We observed that the effect on HFF-1 cell viability at 200 μM TA or EG was 58 and 70%, respectively (Figure 1C). Whereas in Hep3B cells, it was 20 and 40%, respectively. This indicates that HFF-1 cells are much less sensitive than HCC Hep3B cells. A similar effect was observed with PTX co-treatment (Figure 1D).

### 2.2. TA and EG Treatment Potentiates the Effect of PTX on Microtubule Stabilization and the G2/M Phase of the Cell Cycle in HEP3B Cells

**Figure 2:**
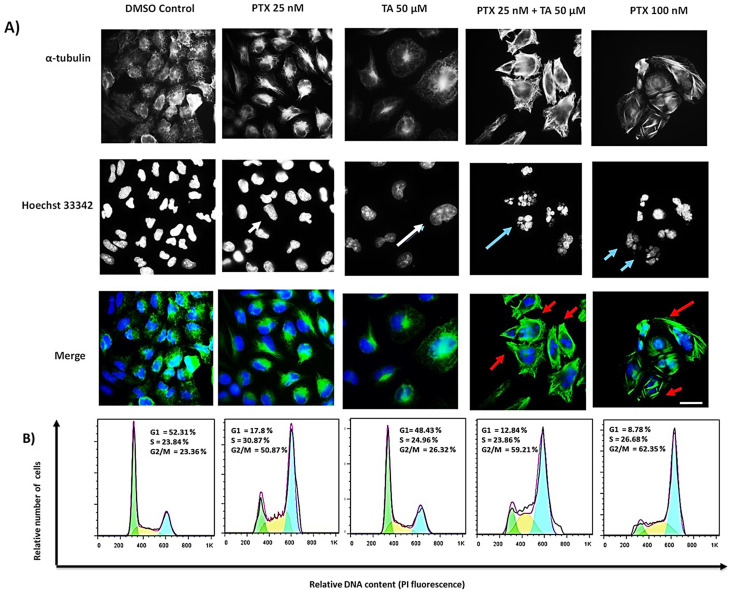
(**A**) Micrographs of Hep3B cells incubated for 24 h with 0.05% DMSO as a negative control, 25 nM PTX, 50 μM TA, 25 nM PTX plus 50 μM TA, and 100 nM PTX. MTs (green) were immunostained with an α-tubulin monoclonal antibody, and DNA (blue) was stained with Hoechst 33342. Scale bar represents 15 μm. (**B**) The effect of the compounds on the cell cycle in Hep3B cells was analyzed by flow cytometry. Cells were treated with 0.05% DMSO as a negative control, 25 nM PTX, 50 μM TA, 25 nM PTX plus 50 μM TA, and 100 nM PTX. Histograms show the percentage of cells in the G0/G1, S, and G2/M phases of the cell cycle.

**Figure 3:**
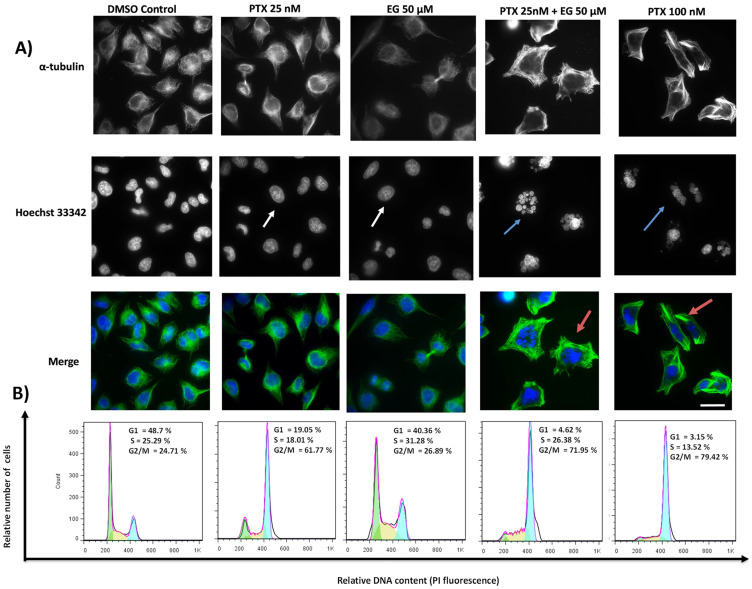
(**A**) Micrographs of Hep3B cells incubated for 24 h with 0.05% DMSO as a negative control, 25 nM PTX, 50 μM EG, 25 nM PTX plus 50 μM EG, and 100 nM PTX. MTs (green) were immunostained with an α-tubulin monoclonal antibody, and DNA (blue) was stained with Hoechst 33342. Scale bar represents 15 μm. (**B**) The effect of the compounds on the cell cycle in Hep3B cells was analyzed by flow cytometry. Cells were treated with 0.05% DMSO as a negative control, 25 nM PTX, 50 μM EG, 25 nM PTX plus 50 μM EG, and 100 nM PTX. Histograms show the percentage of cells in the G0/G1, S, and G2/M phases of the cell cycle.

To verify whether we were indeed observing an enhancement of the effect of PTX, its effect on microtubules (MTs) was verified by immunofluorescence in Hep3B cells after 24 h of treatment. The results are shown in (Figure 2A) for the TA treatments and in (Figure 3A) for the EG treatments, both in combination with PTX.

In the individual treatments of PTX 25 nM and TA 50 μM (concentrations at which individually there was no significant effect on cell viability Figure 1B), the characteristic effect of PTX on microtubule stabilization was not observed (white arrows). However, in the combination of PTX 25 nM and TA 50 μM, stabilized microtubules, the formation of rigid structures at the membrane periphery and perpendicular to the nucleus, were clearly observed (red arrows). Similar to the effect induced by PTX treatment at 100 nM, this effect also induced fragmented nuclei (blue arrows), as is shown in Figure 2A. PTX is a potent microtubule-targeting agent known to cause mitotic cell cycle arrest; so, after observing the combined effect of TA or EG with PTX on microtubule stabilization, we aimed to comparatively analyze the effects of these agents on cell cycle progression in Hep3B cells. The results of the flow cytometry assay showed an arrest at G2/M, where the treatment with 25 nM PTX was 50.87%, which was intensified by the co-treatment 25 nM PTX + 50μM TA to 59.21%. This increase was similar to the 100 nM PTX treatment with 62.35% (Figure 2B).

An enhanced effect of PTX on microtubule stabilization was also observed by co-treatment with EG (red arrows); this combined effect also induced nuclear fragmentation (blue arrows), similar to the effect observed at 100 nM PTX, as shown in (Figure 3A). The results of the flow cytometry assay also showed an arrest at G2/M, where the treatment with 25 nM PTX was 61.77%, which was improved by the co-treatment of 25 nM PTX + 50 μM EG with 71.95%. However, the treatment with PTX at 100 nM was higher at 79.45% (Figure 3B).

In summary, we observed rigid structures in the co-treatment of 25 nM PTX and TA or EG at 50 μM. This indicates a stabilization of the microtubules. This is precisely the combinatorial effect highlighted, suggesting the use of lower concentrations of PTX (in this case, four-fold lower) in combination with TA or EG to obtain an effect comparable to that of PTX at high concentrations (100 nM). Therefore, in view of these results, biochemical assays were performed to verify whether tubulin is the target of EG and TA.

### 2.3. Modulation of Tubulin Assembly by Ligand

**Figure 4:**
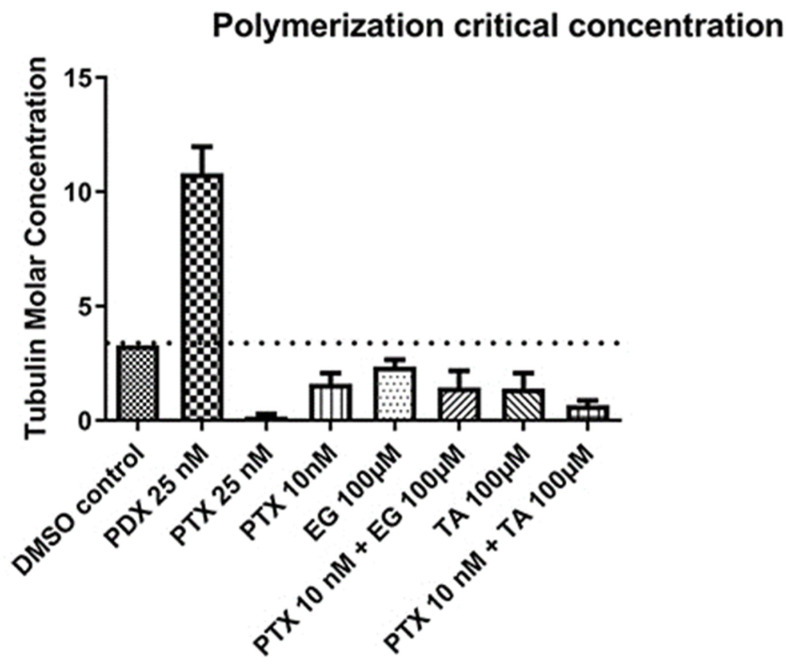
Modulation of tubulin assembly in the presence of the compounds of interest in buffer GAB-1 mM GTP, 6 mM MgCl_2_. Compounds with Cr values above 3.3 μM (dotted line) are considered microtubule-destabilizing agents; compounds with Cr values below 3.3 μM (dotted line) are considered microtubule-stabilizing agents. In total, 25 μM PTX was used as a positive control for the stabilizing agent, and 25 μM PDX (podophyllotoxin) was used as a positive control for the destabilizing agent.

Tubulin assembly can be considered as a non-covalent condensed nucleation polymerization reaction characterized by a cooperative behavior and by the presence of a critical concentration (Cr), below which no significant polymer formation takes place [23]. Therefore, measuring the Cr of the tubulin assembly induced by the ligand/compound allows us to determine the nature of the modulatory effect of the studied compounds on this assembly. In GAB buffer (negative control), tubulin can assemble by itself in the absence of ligand/compound, with a critical concentration of 3.3 ± 0.02 μM (37 °C). A decrease in the Cr value indicates that the added compound acts as a microtubule-stabilizing agent, whereas an increase in this value determines the microtubule-destabilizing capacity of the agent in question [24]. PTX was used as a positive control for microtubule-stabilizing agents. Critical concentrations of ligand-induced tubulin assembly were measured for each compound. The results of tubulin assembly with podophyllotoxin (PDX) (positive control for microtubule destabilizing agent) showed values above 10, higher than the Cr of 3.3 of the negative control (GAB DMSO), clearly indicating its effect as a microtubule destabilizer; on the other hand, with TA, EG, and PTX compounds, Cr values below 3.3 are observed, which is the characteristic effect of a microtubule stabilizing agent. TA at 100 μM showed a similar effect to PTX at 10 nM (Figure 4).

We analyzed the effect of 100 μM TA or 100 μM EG in combination with 10 nM PTX (the concentration at which Cr was not significantly affected when compared to the untreated control) on tubulin assembly. The results show that the effect of 10 nM PTX in combination with 100 μM TA decreased Cr compared to the effect of 25 nM PTX alone, suggesting a potentiating effect on the assembly of PTX to tubulin in the presence of TA. However, combined treatment with 100 μM EG did not alter the Cr level of 10 nM PTX (Figure 4).

### 2.4. Biochemical Characterization of TA and Tubulin Interaction

**Figure 5:**
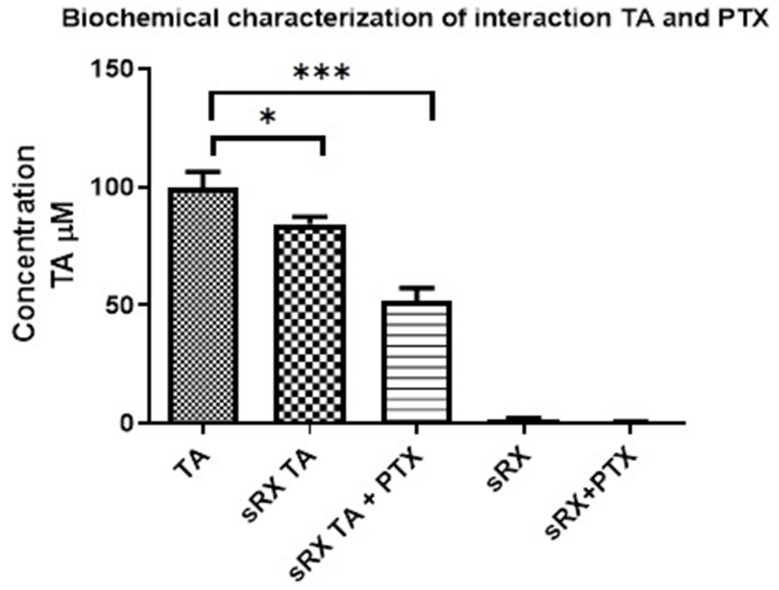
Characterization of the PTX and TA interaction. Absorbance of supernatants from experimental reactions of GAB-tubulin (sRX) plus TA 100 μM, sRX TA, or sRX TA + PTX, with respect to the initial concentration of TA (reference standard), * *p* < 0.05, *** *p* < 0.001.

To understand the effect of the PTX + TA combination on tubulin assembly, a biochemical characterization of the interaction of TA with tubulin, with or without PTX, was performed. The experimental reactions were carried out according to the protocol described in the Materials and Methods Section, where the concentration of free TA (in the supernatant) of each reaction was evaluated. The higher the concentration of free TA (in the supernatant), the lower the interaction with tubulin, and the lower the concentration of free TA, the higher the interaction with tubulin. The results showed that the concentration of free TA decreased significantly in the experimental reaction of tubulin and TA (sRX TA), indicating the physical interaction between tubulin and TA. A similar effect, but of greater intensity, was observed in the sRX TA + PTX reaction, showing that TA can interact with tubulin already assembled with PTX, suggesting different binding sites of TA and PTX on tubulin (Figure 5).

### 2.5. Taxane Displacement Assay

**Figure 6:**
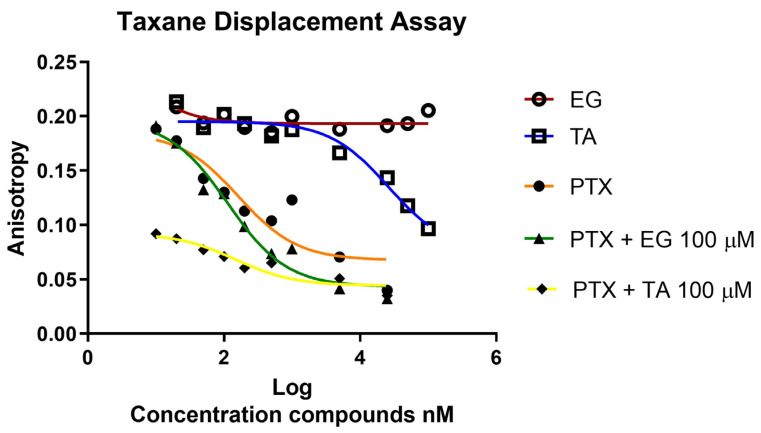
Displacement assay. The displacement is associated with changes in the fluorescence anisotropy values of the Flutax-2 molecule in the presence of its binding site and the EG and AT ligands, individually and in combination with a PTX.

Having confirmed from previous tests that EG and TA act (to a greater or lesser extent) as microtubule-stabilizing agents, both in cells and biochemically characterized in vitro, taxane displacement assays were performed using fluorescence anisotropy. Previous results obtained with EG and TA suggested that they affect microtubule dynamics, so it was of great interest to evaluate whether tubulin is the target of EG and TA. In particular, we investigated whether EG and TA displace Flutax-2 (fluorescent PTX) from its tubulin binding site. PTX was used as a positive control, and an evident displacement was observed with a decrease in fluorescence levels. For EG, the fluorescence levels observed remained constant in the EG treatments, indicating that EG does not displace PTX from its tubulin binding site. However, with respect to TA, it was observed that at concentrations of 1, 5, 25, and 100 μM, there was a decrease in fluorescence levels associated with a displacement of Flutax-2 (Figure 6).

Therefore, we were interested in analyzing the combined effect of TA + PTX and EG + PTX. These results showed that for PTX with TA (yellow line with black diamonds, Figure 6), the displacement was higher compared to the effect of PTX alone (orange line with circles), suggesting an enhanced effect of PTX on displacement in combination with TA. Meanwhile, the combined effect of EG + PTX showed slight changes compared to PTX alone (Figure 6).

### 2.6. Molecular Docking of TA and EG to Tubulin

**Figure 7:**
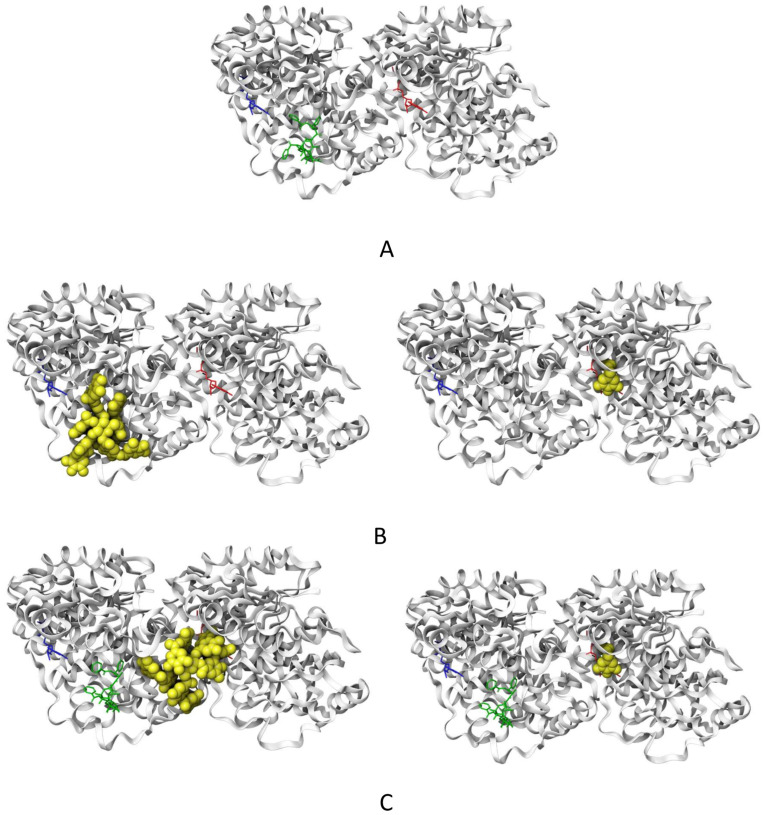
Molecules tested and their interaction site on tubulin. Tubulin is shown in the white cartoon representation (α-tubulin on the right and β-tubulin on the left). GTP is shown in red sticks and GDP in blue sticks. (**A**) PTX binding site (green sticks). (**B**) Position of tannic acid (yellow spheres, **left**) and ethyl gallate (yellow spheres, **right**) obtained from the docking in the absence of PTX. (**C**) Position of tannic acid (yellow spheres, **left**) and ethyl gallate (yellow spheres, **right**) obtained from the docking in the presence of PTX.

Three ligands were studied on the tubulin dimer (PTX, TA, and EG). The PTX molecule is the crystallographic ligand present in the 1JFF structure [25]. To validate our docking procedure, PTX was docked to tubulin in 100 blind docking experiments. Blind docking was chosen due to the lack of information on the binding sites for the other ligands. From these studies, we found that PTX redocking produced structures with an RMSD of 2.2 +/− 0.1 Å compared to the crystallographic structure. Normally, RMSD values below 2 Å are expected from a redocking study. Based on these results, we are confident that the docking methodology used here can reproduce an experimental PTX conformation. TA and EG were docked to tubulin in the same way as PTX, in the presence and absence of PTX. These experiments were performed to explore another possible binding site for both ligands besides the canonical PTX binding site. A representative result of these experiments is shown in Figure 7. The affinity energies obtained in this analysis are shown in Table 1.

The results obtained show that PTX has a lower affinity energy (indicating a better interaction with tubulin Figure 7A) with respect to TA and EG. TA was found at two different sites (cluster 1 and cluster 2). One of them (cluster 1) was the same binding site as PTX (Figure 7B, left). The second one was found in the intermediate region between the α- and β-tubulin (Figure 7C, left). In the presence of PTX, TA was found in the intermediate region (Figure 7C, left). Based on the predictions, TA could have two possible binding sites, the first belonging to the PTX site and the second between the two tubulin dimers. On the other hand, EG was found in a pocket close to the GTP molecule in the presence and absence of PTX. The affinity energies for TA and EG in the presence of PTX are shown in Table 2.

**Figure 8:**
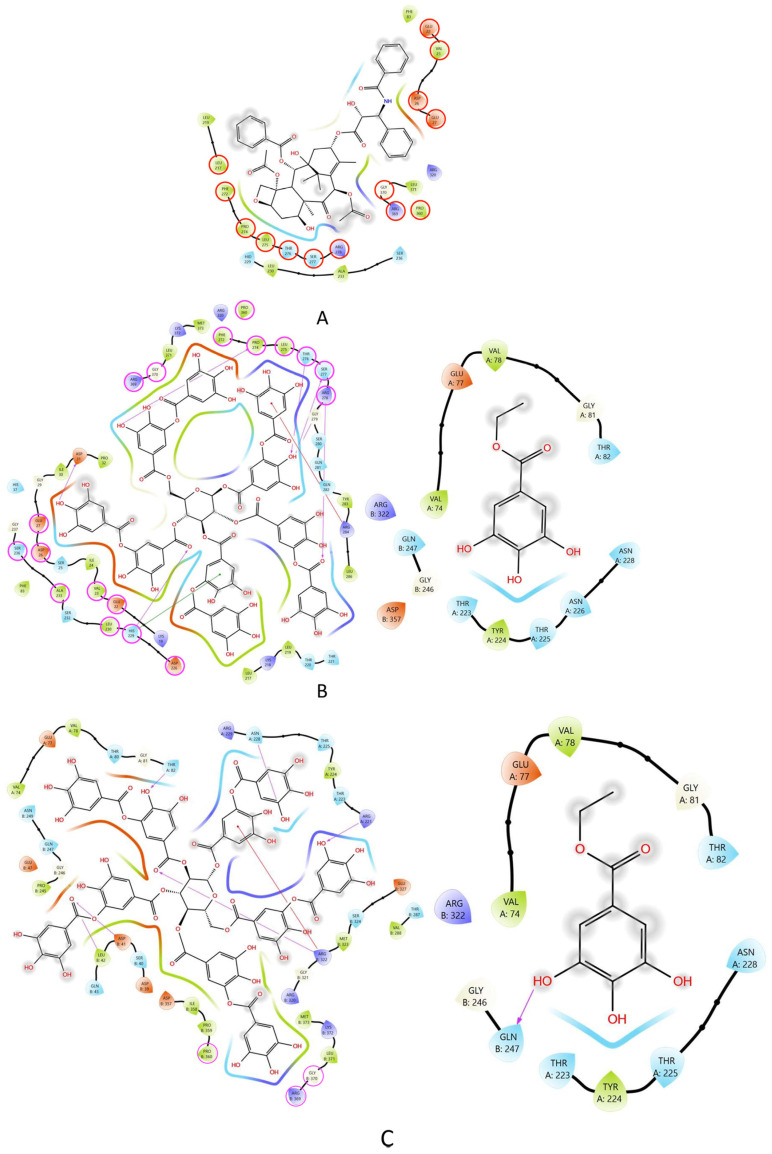
Interaction maps of the compounds tested in this study. (**A**) PTX, red circles represent the conserved amino acids from the interactions observed in the crystallographic structure 1JFF. (**B**) TA (**left**), pink circles indicate the amino acids shared with PTX and EG (**right**). (**C**) Interactions identified between TA (**left**) and EG (**right**) with tubulin in the presence of PTX. Pink circles highlight the amino acids that interact with PTX in the crystallographic structure 1JFF.

The ligand–protein interaction map shows that the molecular docking of PTX with tubulin (Figure 8A) recapitulates 73.7% of the interactions found in the crystallographic structure [25]. On the other hand, the molecular docking of TA without PTX binds to the same site as PTX, reproducing most of the crystallographic interactions (94.7%) (Figure 8B). Furthermore, TA, which has several hydrogen bond donor and acceptor groups, interacts with several amino acids using these groups. When docking TA in the presence of PTX, it binds to the interface between α- and β-tubulin, and due to its size, it can interact with amino acids belonging to the tubulin–tubulin binding site (15.8%) (Figure 8C).

To ensure that our procedure reproduces the crystallographic conformation, we obtained and tabulated the residues in contact with PTX on 1JFF and those reported in other studies [26,27] (Table 3). As mentioned above, our procedure was able to reproduce crystallographic interactions with GLU22, VAL23, ASP26, GLU27, LEU217, HIS 229, LEU230, ALA233, SER236, PHE272, PRO274, LEU275, THR276, SER 277, and ARG278. In addition, PTX redocked interacted with residues reported to interact with other ligands (Table 3).

### 2.7. Effect of TA, EG, and PTX on Protein Kinase Activity

**Figure 9:**
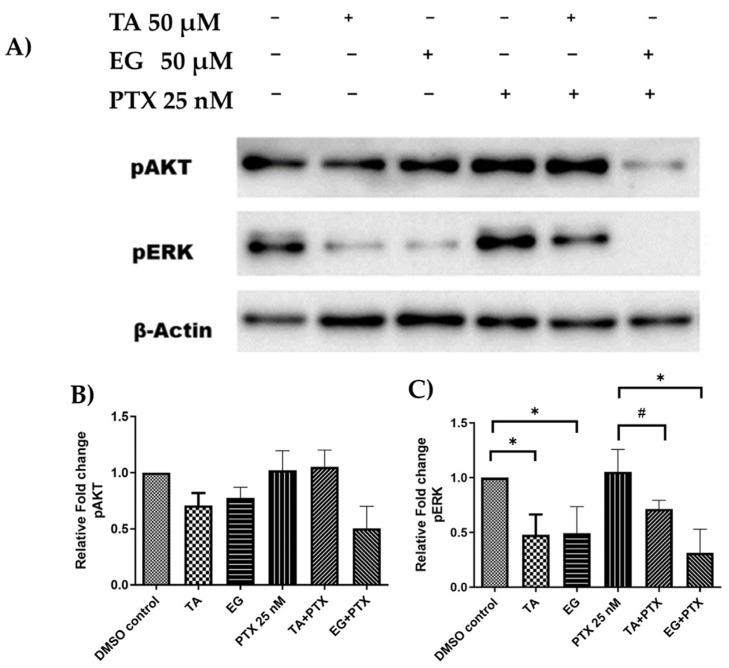
Protein kinase activation. (**A**) Western blot images show the relative phosphorylation/activation of Akt (p-Akt) and ERKs (p-ERK) in untreated Hep3B cells (control) and cells treated for 24 h with TA, EG, and PTX, alone and in combination. β-actin was used as a control for gel loading. TA, tannic acid; EG, ethyl gallate; PTX, paclitaxel. Blots are representative of one of two experiments. Quantification of the relative expression was performed by densitometric analysis for (**B**) pAKT and (**C**) pERK. Data were normalized to the loading control (β-actin). * *p* ≤ 0.05, ^#^ *p* = 0.05.

In the search for TA and EG targets involved in potentiating the effect of PTX in combination with both, the effect of the compounds on the expression of the protein kinases pAkt and pERK was evaluated (Figure 9). These protein kinases restrict cell death (apoptosis) and promote cell proliferation and cell cycle progression. Previous studies have shown that PTX stimulates increased levels of pAkt and pERKp, activating their function and promoting therapeutic resistance to PTX [28,29]. Therefore, the effect of EG and AT, individually and in combination with PTX, on pERK and pAKT levels in Hep3B cells was evaluated.

The results showed a slight effect of TA and EG on pAKT inhibition; however, they strongly reduced pERK levels compared to the control. The combination of EG with PTX significantly decreased the expression/phosphorylation-inducing effect of PTX on pERK and pAKT levels (Figure 9A,B). Taken together, these results suggest that EG, but not TA, negatively regulates AKT and ERK activation under the experimental conditions used in this study. However, to confirm and better understand these results, further studies are needed.

## 3. Discussion

Microtubules are highly dynamic assemblies of tubulin dimers that polymerize and depolymerize during mitosis, the process by which cells divide. Different cellular regulators control microtubule dynamics. However, some drugs and natural compounds can inhibit microtubule polymerization, such as vinca alkaloids [30], Taxol (brand name of Paclitaxel), Taxotere, eleutherobins, epothilones, and laulimalide [31]. Both effects block mitosis and consequently induce cell death by apoptosis.

Microtubule stabilization by binding of PTX (Paclitaxel) to β-tubulin is the most widely accepted mechanism of action. Once bound to taxanes, microtubules cannot be disassembled, so static polymerization disrupts the normal mitotic process, disrupting cells in the G2M phase and ultimately inducing apoptotic cell death [32,33]. However, resistance to PTX often arises through multiple mechanisms after prolonged exposure [34].

In the present study, we investigated the ability of tannic acid (TA) and ethyl gallate (EG) to potentiate the antineoplastic effect of PTX in Hep3B cells. Our cytotoxicity results showed that PTX + TA co-treatment significantly reduced cell viability, while PTX + EG had a lesser effect, both compared to PTX alone. In a previous study, we demonstrated that TA and EG exerted cytotoxicity effects on various cancer lines, inducing cell cycle arrest and apoptosis [22]. Therefore, we investigated the potentiating effect on microtubule stabilization to understand the possible mechanism of action. We observed that the effect of PTX + TA and PTX-EG on microtubules induces rigid structures perpendicular to the nucleus, comparable to the effect of PTX alone at a high concentration (100 nM), in addition to inducing cell arrest in the G2/M phase and nuclear fragmentation. These results correlated strongly with the biochemical affinities observed for PTX + TA in modulating tubulin assembly as a stabilizing agent, the direct interaction of TA with tubulin, and the potentiation of PTX activity in combination. Furthermore, the ability of TA, but not EG, to displace Flutax-2 (fluorescent PTX) from microtubules was demonstrated. In addition, the molecular docking study suggests that TA can bind to tubulin already bound to PTX in cluster 2, suggesting a synergistic effect between TA and PTX on microtubule stabilization. A recent study with two ligands, taxol and taxotere, induced collective changes in the structure and dynamics of the α,β-tubulin dimer; these changes increased the coherence between the communities of residues on the two opposite faces of the β-subunit; this likely creates indirect cooperativity between these structural regions, which would be part of the molecular basis of the ligand over-stabilization on microtubules observed in that study [35]. Based on our results, we propose that TA binds directly to the α, β-tubulin dimer and also assembles when tubulin is bound to PTX. This binding stabilizes microtubules and induces cell arrest at the G2/M phase. Due to the binding of TA in cluster 2, it is proposed that this binding causes over-stabilization, which would be the mechanism by which the effect of PTX is enhanced in co-treatment.

Although EG did not interact directly with tubulin in our biochemical experiments or in molecular docking, the effect of EG on microtubule dynamics is clear, in addition to its enhancing effect on PTX. Therefore, in search of its target, we evaluated its effect on regulatory proteins of members of the MAPs family. It has been documented that PTX increases pERK and pAKT levels, which is a characteristic effect of drugs targeting microtubules and is associated with modulation of the MAPs/ERK and AKT signaling pathways [14,29,36,37,38]. Our results showed that EG, TA, and, in particular, the co-treatment of PTX + EG significantly reduced the levels of pERK. Indeed, it has been widely studied that ERK1/2 promotes cell survival by enhancing the effect of anti-apoptotic molecules, for example, Mcl-1, a member of the BCL2 family, which is phosphorylated at threonine 163 by ERK1/2, thereby increasing its stability and enhancing its anti-apoptotic activity [39]. This suggests that the EG-induced cellular effect may be related to reduced pERK activity rather than direct binding to microtubules, thereby reducing anti-apoptotic and proliferative effects. However, further experiments are required to support this potential effect.

## 4. Materials and Methods

### 4.1. Cell Culture

Hep3B (Hepato-Cellular Carcinoma) cells and HFF-1 (Human Foreskin Fibroblasts) cells obtained from ATCC (American Type Culture Collection, Manassas, VA, USA), were cultured in DMEM medium (Invitrogen, Thermo Fisher Scientific, Inc., Waltham, MA, USA) supplemented with 10% fetal bovine serum (SFB, Invitrogen) and 2 mM glutamine, and all cultures were incubated at 37 °C in a 5% CO_2_ atmosphere.

### 4.2. Cell Cytotoxicity Assay

Initially, 10,000 Hep3B cells were seeded in 0.08 mL of culture medium per well in 96-well plates. HFF-1 cells were used as a non-cancerous control. The same cell density was used. The next day, Hep3B cells were treated with successive dilutions of EG or AT (from 25 μM to 200 μM), PTX (25 nM to 200 nM) and the combination PTX plus EG 50 μM or TA 50 μM. While the HFF-1 cells were treated with successive dilutions of EG or AT (from 25 μM to 800 μM), PTX (25 nM to 400 nM), and the combination PTX plus EG 50 μM or TA 50 μM. All treatments were carried out for 24 h, after which an MTT assay [40] was performed to assess cell viability. Each well received 20 μL of 2.5 mg/mL MTT (3-(4,5-dimethylthiazol-2-yl)-2,5-diphenyltetrazolium bromide), incubated for 4 h at 37 °C, and then treated with 0.1 mL of MTT solubilizer (10% SDS, 45% dimethylformamide [pH 5.5]). Plates were incubated again overnight at 37 °C to dissolve the blue formazan precipitate, and absorbance was measured at 595/690 nm in an automated Multiscan microplate reader. Control wells containing cell-free medium were used as blanks. The MTT response is expressed as a percentage of control (untreated) cells.

### 4.3. Indirect Immunofluorescence

In total, 5.0 × 10^4^ Hep3B cells were added to 24-well culture plates with 12 mm round coverslips and allowed to attach overnight at 37 °C in 5% CO_2_. Cells were treated with EG (50 μM), TA (50 μM), PTX (25 nM), and a combination of PTX 25 nM plus EG 50 μM or TA 50 μM or vehicle (DMSO) for 24 h, with a maximum of 0.5% *v*/*v* DMSO. Adherent Hep3B cells were permeabilized with Triton X-100 and fixed with 3.7% formaldehyde, as previously described [24,41]. After washing with PBS, the coverslips were incubated with the monoclonal antibody against α-tubulin DM1A (1/400 dilution) for 1 h at 37 °C, washed twice and incubated with FITC goat anti-mouse immunoglobulins (1:200 dilution). Finally, after appropriate washes with PBS, the Hoechst 33342 DNA marker was incubated at a 1:100 dilution in PBS for 30 min. The treated coverslips were mounted on conventional slides, examined, and photographed using a Zeiss Axioplan epifluorescence microscope (Zeiss, Jena, Germany), and images were recorded using an ORCH-FLASH 4.0 cooled CCD camera.

### 4.4. Cell Cycle Analysis

Flow cytometry and propidium iodide (IP) DNA detection were used to assess the effect of the treatments on cell cycle progression. Hep3B cells (300,000 per mL) were incubated with EG 50 μM, TA 50 μM, PTX 25 nM, PTX 100 nM, and the combination of PTX 25 plus EG 50 μM or TA 50 μM or DMSO (not more than 0.5% *v*/*v*) for 20 h. Cells were fixed with 70% ethanol, treated with RNase, and stained with IP as previously described [24] and analyzed on a Coulter Epics XL flow cytometer (Beckman Coulter EPICS XL MCL (Beckman Coulter, Fullerton, CA, USA). Two independent experiments were performed.

### 4.5. Ligand-Induced Tubulin Assembly

Critical concentrations for ligand-induced tubulin assembly were measured as previously [24]. Experimental reactions were carried out in a final volume of 200 μL with a tubulin concentration of 20 μM and excess compounds (25 or 100 μM, depending on the experiment). These reactions were incubated for 45 min at 37 °C in a dry heat thermoblock. The microtubules formed were pelleted by centrifugation for 20 min at 50,000 rpm in a TLA 100 rotor preheated to 37 °C using a Beckman Optima TLX centrifuge. The supernatants were carefully removed, and the pellets were resuspended in 10 mM sodium phosphate, 1% SDS (pH 7.0). The pellets and supernatants were diluted 1:10 in the same buffer, and their concentrations were measured fluorometrically using a Fluorolog 3 spectrofluorimeter (excitation wavelength of 285 nm and emission wavelength of 320 nm with 2 and 5 nm slits, respectively) [42].

### 4.6. Biochemical Characterization of TA and Tubulin Interaction

In order to evaluate the effect observed in the displacement assay, it was proposed to carry out the biochemical characterization of the interaction of AT with PTX, for which the following experimental reactions were carried out in a final volume of 200 μL, with a tubulin concentration of 20 μM and the respective compound to be evaluated, 5 experimental reactions described below: (a) DMSO (experimental control of tubulin assembly); TA (100 μM), (b) TA(100 μM) + PTX (25 μM); performed in a final volume of 200 μL, with a tubulin concentration of 20 μM. These reactions were incubated for 45 min at 37 °C in a thermoblock. The microtubules formed during this time were pelleted by centrifugation at 50,000 rpm for 20 min. The supernatants were carefully removed and analyzed by spectrophotometry. The supernatants were carefully removed and analyzed by spectrophotometry at 300 nm to verify their absorbance and compare it with a standard of known concentration and, in relation to this, to analyze if the concentration of the compounds under study decreases, which would be an indication to know if the compound binds to tubulin because if so, the tubulin would remain in the pellet after centrifugation and if the compound binds to tubulin, therefore the concentration of the supernatant of the compound of interest should decrease.

### 4.7. Taxane Displacement Assay

In order to determine if the evaluated tannic acid (TA), ethyl gallate (EG), binds to the paclitaxel site in the stabilized microtubules, the change in fluorescence anisotropy values of the flutax-2 molecule was measured in the presence of its binding site and ligands [42]. A mixture of 50 nM flutax-2 and 50 nM microtubule-stabilized paclitaxel sites were prepared in GAB-1 mM GTP. Then, 200 μL of the mixture were added to each well of a 96-well plate, avoiding the edges of the wells. Onto this mixture, increasing concentrations of the ligand challenge (up to 100 μM) were added. The reaction mixtures were incubated on an orbital shaker for 1 h, and the anisotropy values of each well were measured at different temperatures using an Appliskan (Thermo) plate reader. The samples were excited with pulses of vertically polarized light (using a 485P filter, with a bandpass of 480–492 nm), and the emission was analyzed simultaneously with horizontal and vertical polarized filters (using a 520P filter, with a bandpass of 515–550 nm). The gain of both channels is adjusted based on the anisotropy of free flutax-2 (r = 0.055 and polarization = 0.080, in GAB buffer at 25 °C) in wells containing flutax-2 without microtubules. Blanks were measured in wells containing microtubules without flutax-2, and fluorescence intensity values were restored for each well.

### 4.8. Molecular Docking

Tubulin structure was obtained from the Protein Data Bank (PDB ID: 1JFF) [25]. Protein–ligand docking studies were performed with PTX, TA (PUBCHEM ID: 16129778) and EG (PUBCHEM ID: 13250). The PTX molecule was redocked to the tubulin structure to validate our molecular docking studies. PTX and ions were removed from 1JFF, and the final structure was energy minimized on Chimera UCSF [43] with 100 steepest descent steps and 10 conjugate gradient steps prior to the molecular docking procedure. The best conformer of all ligands was obtained using MarvinSketch 22.22, Chemaxon [44].

One hundred independent blind docking studies were realized with each ligand. The grid box was located at (17.65, −4.49, and 4.86) with a size of 120.91 × 60.51 × 63.88 Å^3^. Autodock vina 1.1.2 [45] was used for molecular docking. We clustered all results by RMSD and presented only the most populated cluster for each ligand (unless otherwise indicated). Figures and interaction maps were obtained using Maestro 2023.3 from Schrodinger [46].

### 4.9. Western Blotting

Rabbit polyclonal antibodies (pAb): phospho-p38-MAPK (Thr180/Tyr182), phospho-Akt (Ser473), phospho-p44/42 MAPK (Erk1/2), and Thr202/Tyr204, were purchased from Cell Signaling Technology (Danvers, MA). Secondary antibodies were purchased from DAKO Diagnostics, S.A. (Barcelona, Spain). For Western, the following procedures were used: cells were harvested 24 h after treatment, treated with lysis buffer, and protein concentration was determined. Proteins were solubilized in 2X Laemmli buffer with β-mercaptoethanol, heated at 95 °C for 3 min, and loaded on a 10% SDS-PAGE gel. The transfer was performed using the Trans-Blot^®^ Turbo™ transfer system on a PVDF membrane, after which the membrane was blocked for 1 h with 5% milk in TBS-Tween 20, washed as appropriate and incubated overnight at 4 °C with the primary antibody. The antibodies used were Phospho-Akt (Ser473) 1:200 dilution; Phospho-p44/42 MAPK (Erk1/2) Thr202/Tyr204 dilution 1:1000.

The membrane was washed twice in TBS-Tween followed by incubation for 1 h at room temperature with the appropriate secondary antibody, anti-rabbit IgG conjugated to HRP at a dilution of 1:2000. The NZY Supreme ECL HRP substrate (NZYTech, Lda., Lisbon, Portugal) was used as a developer and the ChemiDoc touch imaging system (Bio-Rad Laboratories, Inc., Hercules, CA, USA) was used for protein visualization. β-Actin was used as a loading control at a dilution of 1:2000.

## 5. Conclusions

Our results support that tannic acid and ethyl gallate are potential chemotherapeutic agents due to their potentiating effect on paclitaxel. This may be therapeutically beneficial in HCC, particularly when targeted therapy may not be sufficiently effective.

## Figures and Tables

**Table 1:** Ligand binding affinity energy (kcal/mol) with tubulin in the absence of PTX.

Ligand	Affinitty Energy (Kcal/mol)
PTX (62%) ^a^	−9.0 +/− 0.2
TA (cluster 1) (30%) ^a^	−8.6 +/− 0.5
TA (cluster 2) (20%) ^a^	−8.4 +/− 0.4
EG (77%) ^a^	−6.5 +/− 0.1

^a^ The number in parentheses indicates the percentage of structures that correspond to that cluster.

**Table 2:** Ligand binding affinity energy (kcal/mol) with tubulin in the presence of PTX.

Ligand	Affinitty Energy (Kcal/mol)
Tannic Acid (23%) ^a^	−8.7 +/− 0.6
Ethyl Gallate (68%) ^a^	−6.5 +/− 0.1

^a^ The number in parentheses indicates the percentage of structures that correspond to that cluster.

**Table 3:** Tubulin/residue interactions found on 1JFF (crystal structure), PTX redocking, and distinct combinations studied here. Ligand binding sites are shown for comparison (last column).

Residue	Tubulin	1JFF	REDOCKING	TA	TA + PTX	EG	EG + PTX	Ligands [27]
VAL74	α				*	*	*	
GLU77	α				*	*	*	
VAL78	α				*	*	*	
THR80	α				*			
GLY81	α				*	*	*	
THR82	α				*	*	*	
ARG221	α				*			
THR223	α				*	*	*	
TYR224	α				*	*	*	
THR225	α				*	*	*	
ASN226	α					*		
ASN228	α				*	*	*	
ARG229	α				*			
LYS19	β			*				L2
GLU22	β	*	*	*				L2
VAL23	β	*	*	*				L2, L3, PTX
ILE24	β			*				
SER25	β			*				
ASP26	β	*	*	*				PTX
GLU27	β	*	*	*				L2, L3
GLY29	β			*				
ILE30	β			*				
ASP31	β			*				
PRO32	β			*				
HIS37	β			*				
ASP39	β				*			
SER40	β				*			
ASP41	β				*			
LEU42	β				*			
GLN43	β				*			
GLU47	β				*			
GLU55								PTX
PHE83	β		*	*				
CYS213	β	*						L1, L2, L3
LEU217	β	*	*	*				L1, L2, L3, PTX
LYS218	β			*				
LEU219	β		*	*				L1, L2, L3, PTX
THR220	β			*				
THR221	β			*				
ASP226	β	*		*				L1, L2, L3, EPOA
ASN228	β							ZMP
HIS229	β	*	*	*				L1, L2, L3, PTX, ZMP
LEU230	β	*	*	*				L1, L2, L3, PTX
SER232	β			*				L2
ALA233	β	*	*	*				L1, L2, L3, PTX
SER236	β	*	*	*				L2, L3, PTX
GLY237	β			*				L2
PRO245	β				*			
GLY246	β				*	*	*	
GLN247	β				*	*	*	
ASN249	β				*			
PHE272	β	*	*	*				L1, L2, L3, PTX
PRO274	β	*	*	*				L1, L2, L3, PTX
LEU275	β	*	*	*				L1, L2, L3, PTX
THR276	β	*	*	*				L1, L2, L3, PTX, EPOA, ZMP
SER277	β	*	*	*				L1, L2, L3, PTX
ARG278	β	*	*	*				L1, L2, L3, PTX
GLY279	β			*				
SER280	β			*				
GLN281	β			*				PTX, EPOA
GLN282	β			*				L3
TYR283	β			*				
ARG284	β			*				PTX
LEU286	β			*				PTX
THR287	β				*			
VAL288	β				*			
LEU291	β							PTX
GLN294	β							PEL
PHE296	β							LAU, PEL
ASP297	β							PEL
ALA298	β							PEL
PRO307	β							PEL
ARG308	β							LAU, PEL
TYR312	β							LAU, PEL
ARG320	β		*	*	*			L2, L3
GLY321	β				*			
ARG322	β				*	*	*	
MET323	β				*			
SER324	β				*			
GLU327	β				*			
VAL335	β							LAU, PEL
ASN339	β							LAU, PEL
SER341	β							LAU
TYR342	β							LAU, PEL
PHE343	β							LAU
ILE347	β							LAU
ASP357	β				*	*		
ILE358	β				*			
PRO359	β				*			
PRO360	β	*	*	*	*			L2, L3, PTX
ARG369	β	*	*	*	*			L1, L2, L3, PTX
GLY370	β	*	*	*	*			L1, L2, L3, PTX
LEU371	β		*	*	*			L1, L2, L3, PTX
LYS372	β			*	*			
MET373	β			*	*			
SER374	β							PTX

L1 = baccatin III, L2 = 2a, L3 = 2b, PTX = Paclitaxel, EPOA = Epothilone A, ZMP = Zampanolide, LAU = Laulimalide (β subunit), PEL = Peloruside A (β subunit). * = interactions found in this study.

## Data Availability

Data is contained within the article.
